# Relationship between ascending thoracic aortic diameter and blood pressure, a Mendelian randomization study

**DOI:** 10.1161/ATVBAHA.122.318149

**Published:** 2023-01-05

**Authors:** John DePaolo, Michael G. Levin, Catherine Tcheandjieu, James Priest, Dipender Gill, Stephen Burgess, Scott M. Damrauer, Julio A. Chirinos

**Affiliations:** 1 Department of Surgery, Perelman School of Medicine, University of Pennsylvania, Philadelphia, PA, USA; 2 Division of Cardiovascular Medicine, Department of Medicine, Perelman School of Medicine, University of Pennsylvania, Philadelphia, PA, USA; 3 Gladstone Institute of Data Science and Biotechnology, Gladstone Institutes, San Francisco, CA, USA; 4 Department of Epidemiology and Biostatistics, University of California San Francisco, San Francisco, CA, USA; 5 Department of Pediatrics – Division of Pediatric Cardiology, Stanford University School of Medicine, Stanford, CA, USA; 6 Chief Scientific Advisor Office, Research and Early Development, Novo Nordisk, Copenhagen, Denmark; 7 Department of Epidemiology and Biostatistics, School of Public Health, Imperial College London, UK; 8 MRC Integrative Epidemiology Unit, University of Bristol, Bristol, UK; 9 Department of Public Health and Primary Care, University of Cambridge, Cambridge, UK; 10 Corporal Michael Crescenz VA Medical Center, Philadelphia, PA, USA; 11 Department of Genetics, Perelman School of Medicine, University of Pennsylvania, Philadelphia, PA, USA

**Keywords:** aorta, blood pressure, aortic aneurysm, aortic dilation, Mendelian randomization, genomics

## Abstract

**Background:**

Observational studies identified elevated blood pressure (BP) as a strong risk factor for thoracic aortic dilation, and BP reduction is the primary medical intervention recommended to prevent progression of aortic aneurysms. However, while BP may impact aortic dilation, aortic size may also impact BP. The causal relationship between BP and thoracic aortic size has not been reliably established.

**Methods:**

Genome-wide association studies summary statistics were obtained for BP and ascending thoracic aortic diameter (AscAoD). Causal effects of BP on AscAoD were estimated using two-sample Mendelian randomization (MR) using a range of pleiotropy-robust methods.

**Results:**

Genetically-predicted increased systolic BP (SBP), diastolic BP (DBP), and mean arterial pressure (MAP) all significantly associate with higher AscAoD (SBP: β estimate = 0.0041 mm/mmHg, 95% confidence interval [CI] 0.0008 to 0.0074, p = 0.02, DBP: β estimate = 0.0272 mm/mmHg, 95%CI 0.0224 to 0.0320, p < 0.001, and MAP: β estimate = 0.0168 mm/mmHg, 95%CI 0.0130 to 0.0206, p < 0.001). Genetically-predicted pulse pressure (PP), meanwhile, had an inverse association with AscAoD (β estimate = -0.0155 mm/mmHg, 95%CI -0.0213 to -0.0096, p < 0.001). Multivariable MR analyses showed that genetically-predicted increased MAP and reduced PP were independently associated with AscAoD. Bidirectional MR demonstrated that genetically-predicted AscAoD was inversely associated with PP (β estimate = -2.0721 mmHg/mm, 95%CI -3.1137 to -1.0306, p < 0.001) and SBP (β estimate = -1.2878 mmHg/mm, 95%CI -2.3533 to -0.2224, p = 0.02), while directly associated with DBP (0.8203 mmHg/mm, 95%CI 0.2735 to 1.3672, p = 0.004).

**Conclusions:**

BP likely contributes causally to ascending thoracic aortic dilation. Increased AscAoD likely contributes to lower SBP and PP, but not DBP, consistent with the hemodynamic consequences of a reduced aortic diameter.

## Graphical abstract

**Figure:**
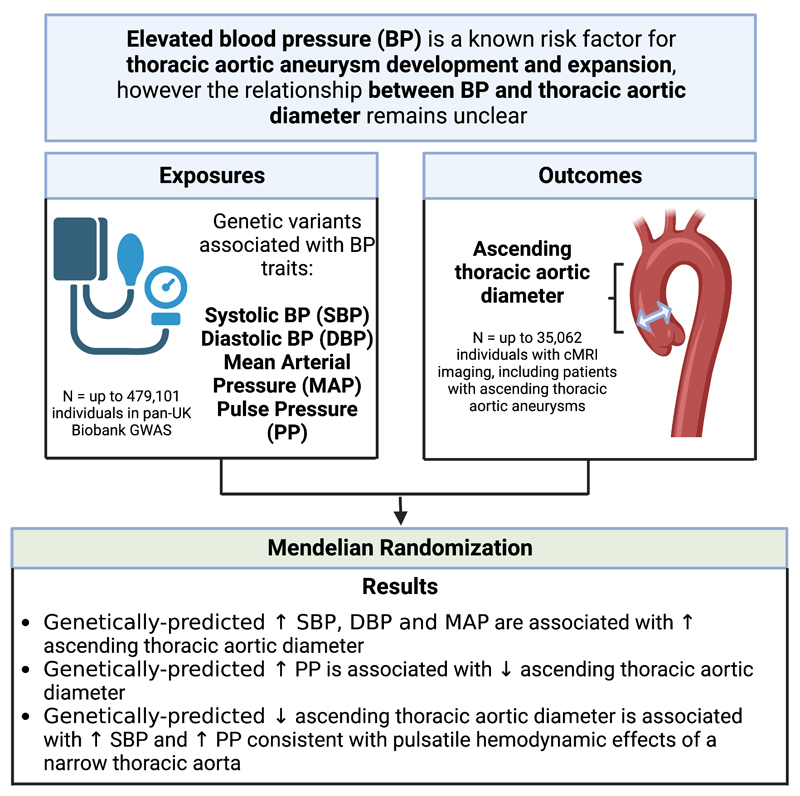
Graphical Abstract created with Biorender.com

## Abbreviations/Acronyms

AscAoD: Ascending aortic diameter
TAA: Thoracic aortic aneurysm
cMRI: Cardiac Magnetic Resonance Imaging
SBP: Systolic Blood Pressure
DBP: Diastolic Blood Pressure
MAP: Mean Arterial Pressure
PP: Pulse Pressure
MR: Mendelian Randomization
UKBB: UK Biobank
MVP: Million Veterans Program
IVW: Inverse-Variance Weighted

## Introduction

The thoracic aorta is the largest vascular structure in the human body, and has important hemodynamic functions beyond its role as a major conduit.^1,2^ The thoracic aorta has a major role in blood pressure (BP) regulation due to its impact on pulsatile hemodynamics, which are in turn influenced by aortic size and wall stiffness.^3^ Conversely, BP is thought to impact the risk of thoracic aortic dilation and thoracic aortic aneurysms (TAA). Additional risk factors include older age, atherosclerosis, smoking, chronic obstructive pulmonary disease (COPD) and a family history of aortic aneurysms.^4^ Currently, medical treatment for prevention of aneurysmal expansion centers around aggressive BP control, assuming a causal impact of BP on aneurysm growth.^5^

Although observational studies have linked hypertension to thoracic aortic aneurysm development and expansion,^6,7^ the causal relationship between BP and thoracic aortic diameter/dilation remains incompletely understood. Several studies indicate that hypertension is associated with increased thoracic aortic diameter; however, these studies may be limited by residual confounding and reverse causality.^8,9^

Our understanding of the genetic architecture of elevated BP has expanded tremendously over the last 15 years in part due to genome-wide association studies (GWAS) identifying single-nucleotide polymorphisms (SNPs) associated with hypertension.^10^ A recently completed trans-ancestry GWAS of systolic BP (SBP), diastolic BP (DBP), mean arterial pressure (MAP), and pulse pressure (PP) identified 535 independent, validated loci among up to 757,601 individuals in the International Consortium for BP and UK Biobank (UKBB).^11^ This study relied on manual and automated BP measurements, and adjusted for antihypertensive medication use.

Another recently completed GWAS of ascending aortic diameter (AscAoD) identified 41 independent, validated loci among UKBB participants utilizing computationally extracted aortic phenotypes from prospectively obtained cardiac MRI (cMRI) images.^12^ Imaging was analyzed using automated segmentation in up to 36,095 patients. The distribution of aortic diameters among the study population was similar to the distribution estimated in the general population. A polygenic risk score was derived from this study, which predicted an increased risk of thoracic aortic aneurysm (OR = 1.50, 95% CI = 1.12-2.01) and subsequent surgical intervention (OR = 1.44, 95% CI = 1.15-1.80).

Mendelian randomization can leverage genetic variation to provide unconfounded estimates of the causal relationship between two traits.^13^ This is because genetic variation is often a product of random allocation of genetic information occurring during meiosis leading to random inheritance of different traits, which precede confounding by various post-conception factors, therefore serving as more reliable instruments for causal inference. Genetic instruments that associate strongly with a particular exposure allow for testing its relationship to an outcome of interest.

In this study, we utilize the two-sample MR to establish the causal relationship between BP and AscAoD, and highlight the intricate bidirectional relationship between BP and aortic dilation.

## Methods

### Study Exposures

Trans-ancestry GWAS of SBP, DBP, MAP, and PP were obtained from the Pan UKBB resource.^11^ These studies included up to 479,101 participants (417,001 European, 8398 Central/South Asian, 6408 African, 2564 East Asian, 1522 Middle Eastern, and 950 American [Hispanic/Latino]). BP measurements were performed manually or in an automated fashion and adjusted for antihypertensive use. Genotyping protocols, quality control details, and summary statistics can be found at https://pan.ukbb.broadinstitute.org/.

To validate our findings, we utilized summary statistics from the trans-ancestry GWAS including up to 318,891 participants of SBP, DBP, and PP from the Million Veterans Project.^14^ The sample size was 91.5% male and enriched for both African-American ancestry and Hispanic ancestry relative to the general US population. The discovery population included 220,501 whites, 59,933 blacks, 21,279 Hispanics, 2,464 Asians, and 2,695 Native Americans. Anti-hypertensive medication use was corrected for and ranged from 31% for Asians to 53% of blacks. These data are available through dbGaP (https://www.ncbi.nlm.nih.gov/gap/) with accession number phs001672.v1.p1.

### Study Outcomes

Ascending thoracic aortic diameter (AscAoD) was obtained from Tcheandjieu *et al*. who quantified ascending aortic diameters from magnetic resonance imaging (MRI) studies obtained from up to 35,062 UKBB participants who had both imaging and genetic data available.^12^ Calculation of AscAoD was performed using transverse (axial) plane images of the thorax at approximately the T4 level and corresponding to the bifurcation of the main pulmonary artery.^15^ Notably, this study did not exclude aneurysmal thoracic aortas. A discovery GWAS was performed with a subset of the participants that included 32,221 white Europeans, 262 African-Afro-Caribbeans, 441 South Asians, and 133 East Asians. Summary statistics of this study are available from the Open GWAS catalog (https://www.ebi.ac.uk/gwas/) under accession number GCP000259. All imaging and genetic data of UKBB participants are available upon request from the UKBB organization. Details regarding calculations of the precise diameter are described and publicly available (https://github.com/priestlab/aorta_houghcircle).

To prevent outlying aneurysmal aortas from biasing our findings, we utilized a more recent study by Pirruccello *et al*, that investigated similar cMRI imaging to characterize AscAoD phenotype from up to 42,518 multi-ethnic UKBB participants and excluded all participants with ascending thoracic aortas > 5 cm in diameter.^16^ These data were derived from a deep learning model consisting of a U-Net-derived architecture using ImageNet, as described, and are publicly available at the Broad Institute Cardiovascular Disease Knowledge Portal (https://cvd.hugeamp.org/).

### Mendelian Randomization

Two-sample MR analyses were performed in R using the *TwoSampleMR* package (https://github.com/MRCIEU/TwoSampleMR).^17^ Genetic instruments of BP traits were constructed from respective GWAS studies using variants in linkage equilibrium, physically separate (*r^2^*<0.001, distance=10000 kb; 1000 genome reference panel), and associated with each trait at genome-wide significance (p<5x10^-8^). For bidirectional MR analyses, genetic instruments were constructed for AscAoD using the same procedure. F statistics for each variant were calculated using the formula F=β2/SE^2^. The primary MR analyses used inverse-variance weighted modeling with random effects. The MR-Egger intercept test was used to test for horizontal pleiotropy. Leave-one-out, single-SNP, and funnel-plot diagnostic MR analyses were performed. Weighted median, weighted mode, and simple mode, all of which make different assumptions about the presence of pleiotropy, were used to perform sensitivity analyses.^18^ Multivariable MR was employed to jointly estimate direct effects of BP traits, again using genetic instruments based on variants in linkage equilibrium, physically separate (*r*^2^<0.001, distance=10000 kb), and associated with genome-wide significance (*p*<5x10^-8^), weighted by the effect of each SNP on each exposure.^19^ Direction of effect was assessed using MR-Steiger.^20^

### Statistical Analysis

The primary analysis of the effect of BP on AscAoD was performed using 2-sample MR inverse-variance weighting considering trans-ancestry BP exposures and outcomes. Instruments were filtered to include only those with F statistic > 10 to minimize weak instrument bias. Cochran Q test and *I^2^* were used to assess heterogeneity. All statistical analyses were performed using R version 4.2.1 (R Foundation for Statistical Computing, Vienna, Austria).

## Results

### Effect of Genetically-Predicated BP Variation on AscAoD

**Figure 1:**
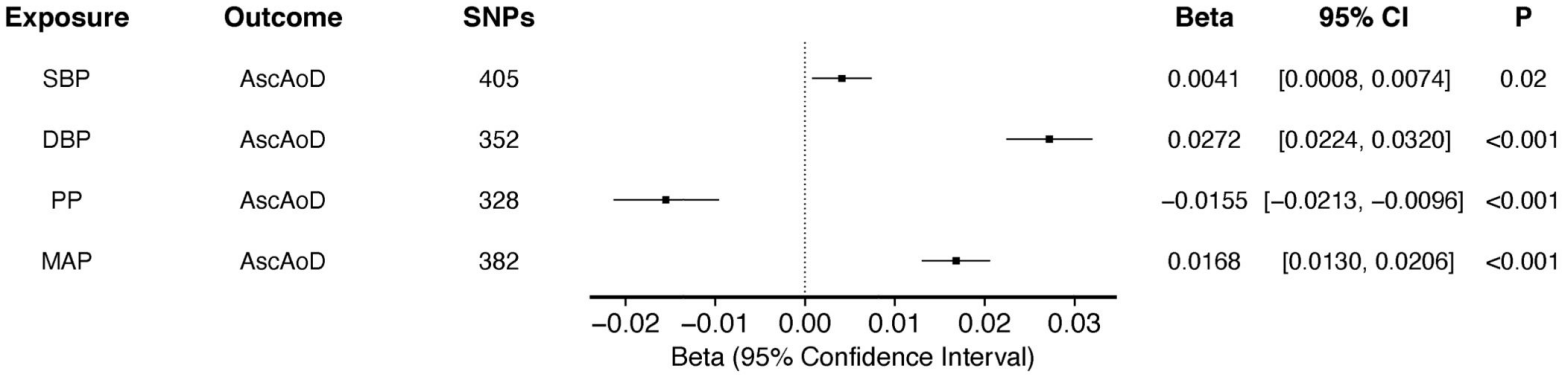
Association between genetically-predicted blood pressure traits and ascending thoracic aortic diameter by univariate two-sample MR using IVW analysis among up to 479,101 participants in the UKBB with BP traits and a cohort of up to 35,062 who underwent MRI for thoracic diameter evaluation. Genetically-predicted SBP (β estimate = 0.0041 mm/mmHg, 95% CI 0.0008 to 0.0074, p = 0.02), DBP (β estimate = 0.0272 mm/mmHg, 95%CI 0.0224 to 0.0320, p < 0.001), and MAP (β estimate = 0.0168 mm/mmHg, 95%CI 0.0130 to 0.0206, p < 0.001) exhibited a positive association with. PP (β estimate = -0.0155 mm/mmHg, 95%CI - 0.0213 to -0.0096, p < 0.001) exhibited a negative association with ascending thoracic aortic diameter.

We performed 2-sample MR using summary statistics from trans-ancestry GWAS to estimate the effect of genetically-predicted changes in SBP, DBP, MAP, and PP on AscAoD. Genetic instruments for these BP traits contained between 328 and 405 independent genetic variants. F statistics ranged from 29.76 – 938.32 (consistent with low risk of weak-instrument bias; Supplementary Tables 1 and 2). Genetically-predicted SBP, DBP, and MAP were positively associated with increased ascending thoracic aortic diameter (Figure 1) in univariate two-sample MR using an inverse-variance weighted (IVW) analyses (SBP: β estimate = 0.0041 mm/mmHg, 95% confidence interval [CI] 0.0008 to 0.0074, p = 0.02, DBP: β estimate = 0.0272 mm/mmHg, 95%CI 0.0224 to 0.0320, p < 0.001, and MAP: β estimate = 0.0168 mm/mmHg, 95%CI 0.0130 to 0.0206, p < 0.001). Genetically-predicted PP, however, was inversely related to AscAoD (β estimate = -0.0155 mm/mmHg, 95%CI -0.0213 to - 0.0096, p < 0.001). The results remained robust in sensitivity analyses using MR methods that make different assumptions about the presence of pleiotropy (Supplementary Table 3).

As the cMRI data from our primary outcomes included frankly aneurysmal thoracic aortas, we employed the same BP exposures in two-sample MR and derived outcome data using summary statistics from a subsequent GWAS by Pirruccello *et al* that utilized a deep learning model to evaluate ascending and descending aortic dimensions for phenotype development and excluded any aorta > 5 cm in diameter (Supplementary Tables 4 and 5).^16^ Consistent with our initial findings, genetically-predicted SBP, DBP, and MAP were positively associated with AscAoD (Figure S1) (SBP: β estimate = 0.0054 mm/mmHg, 95% CI 0.0016 to 0.0093, p = 0.006, DBP: β estimate = 0.0306 mm/mmHg, 95% CI 0.0253 to 0.0360, p < 0.001, MAP: β estimate = 0.0190 mm/mmHg, 95% CI 0.0148 to 0.0232, p < 0.001). Meanwhile, PP was inversely associated with AscAoD (β estimate = -0.0174, 95% CI -0.0243 to -0.0104, p < 0.001).

For comparison between different exposure cohorts, we constructed genetic instruments using summary statistics from trans-ancestry GWAS in a separate cohort of Million Veterans Project (MVP) participants generating alternative exposures.^14^ Notably, genetic instruments for BP traits contained 329 and 316 independent variants for SBP and PP, respectively, but only 67 independent variants for DBP. F statistics ranged from 24.09 - 561.46 (Supplementary Tables 6 and 7). Results were consistent with our primary analysis for genetically-predicted SBP and PP (Supplementary Table 8, Figure S2; SBP: β estimate = 0.0058 mm/mmHg, 95% CI 0.0001 to 0.0115, p = 0.05; PP: β estimate = -0.0159 mm/mmHg, 95% CI -0.0255 to -0.0063, p = 0.002). While the point estimate for DBP exhibited the same direction as in our primary analyses, the confidence intervals were wide and lacked statistical significance (DBP: β estimate = 0.0107 mm/mmHg, 95% CI -0.0207 to 0.0421, p = 0.5).

### Multivariable MR

**Figure 2:**
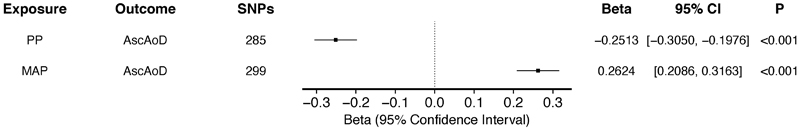
Association between genetically-predicted BP traits and ascending thoracic aortic diameter in multivariate two-sample MR using IVW analysis among up to 479,101 participants in the UKBB with BP traits and a cohort of up to 35,062 who underwent MRI for thoracic diameter evaluation. Genetically-predicted MAP (β estimate = 0.2624 mm/mmHg, 95% CI 0.2086 to 0.3163, p < 0.001) exhibited an independent positive association with ascending thoracic aortic diameter while PP (β estimate = -0.2513 mm/mmHg, 95% CI -0.3050 to -0.1976, p < 0.001) exhibited an independent negative association.

Because BP traits are highly correlated and fundamentally related to MAP (which depends on cardiac output and microvascular resistance) and PP (which depends on stroke volume and arterial stiffness), we performed multivariable MR to investigate the direct effects of MAP and PP (Supplementary Table 9). Both genetically-predicted PP (β estimate = -0.2513 mm/mmHg, 95% CI -0.3050 to -0.1976, p < 0.001) and MAP (β estimate = 0.2624 mm/mmHg, 95% CI 0.2086 to 0.3163, p < 0.001) were independently associated with AscAoD dimension (Figure 2).

### Effect of ascending thoracic aortic diameter on BP

**Figure 3:**
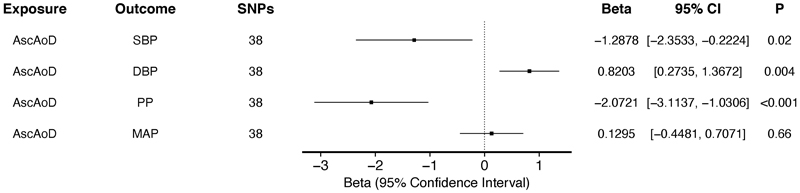
Association between genetically-predicted ascending thoracic aortic diameter and BP traits in two-sample MR using IVW analysis among up to 35,062 participants who underwent MRI for thoracic diameter evaluation and 479,101 participants with BP traits in the UKBB. Genetically-predicted ascending thoracic aortic diameter exhibited a negative association with PP (β estimate = -2.0721 mmHg/mm, 95% CI -3.1137 to -1.0306, p < 0.001) and SBP (β estimate = -1.2878 mmHg/mm, 95% CI -2.3533 to -0.2224, p = 0.02), while having a positive association with increased DBP (β estimate = 0.8203 mmHg/mm, 95% CI 0.2735 to 1.3672, p = 0.004).

To assess whether a concordant relationship exists between AscAoD and BP traits, we performed bidirectional analysis using IVW MR (Supplementary Tables 10 and 11). Genetically-predicted AscAoD was inversely associated with PP (β estimate = -2.0721 mmHg/mm, 95% CI -3.1137 to -1.0306, p < 0.001) and SBP (β estimate = - 1.2878 mmHg/mm, 95% CI -2.3533 to -0.2224, p = 0.02), but positively associated with DBP (β estimate = 0.8203 mmHg/mm, 95% CI 0.2735 to 1.3672, p = 0.004) (Figure 3). The relationship between AscAoD and MAP was not significant (β estimate = 0.1295 mmHg/mm, 95% CI -0.4481 to 0.7071, p = 0.66). These findings indicate that increased AscAoD is negatively associated with systolic hypertension and increased PP. Consequently, these results support a bidirectional relationship that suggests a protective mechanism of aortic dilation against systolic hypertension, however they do not rule out a shared etiology between thoracic aortic dimension/stiffness and BP traits.

## Discussion

This MR study leveraged natural genetic variation to examine the causal relationship between BP and AscAoD. The principal findings were (1) higher genetically-predicted SBP, DBP, and MAP are associated with a larger AscAoD; (2) a higher genetically-predicted PP is associated with a smaller AscAoD; (3) the causal relationships between BP traits and AscAoD were consistent in multivariable MR analyses; and (4) Genetically-predicted AscAoD is inversely associated with SBP ad PP suggesting a protective mechanism of aortic dilation against systolic hypertension (which is consistent with the influence of aortic diameter on pulsatile hemodynamics) and/or a shared etiology between thoracic aortic dilation and SBP and PP. There are several clinically relevant implications from this study.

First, our results support previous observational studies that suggest elevated BP is an independent causal factor to ascending thoracic aortic dilation. Unlike previous observational studies, our MR study leveraged genetic variants as instrumental variables for SBP, DBP, and MAP. As genetic variants are randomly inherited by offspring from parents mimicking randomization of elevated BP among individuals, the MR framework is less susceptible to residual environmental confounding than traditional observational studies.^13^ We also found a strong inverse association between increased PP and AscAoD, supporting previously published studies.^21,22^ Our findings were consistent when excluding frankly aneurysmal aortas (Figure S1) suggesting that this relationship exists in their absence. These results were supported by multivariable MR, demonstrating that BP traits (including PP) associate with ascending thoracic aortic dilation. Overall, the MR findings in this study are consistent with genetically-predicted changes in BP causally effecting ascending thoracic aortic dimension.

We found that genetically-predicted smaller ascending thoracic aorta is inversely related to SBP and PP. This result is consistent with predisposition of a narrow thoracic aorta causally contributing to increased pulse pressure, which is the basis of isolated systolic hypertension, the most common form of hypertension in middle-aged and older adults.^3,23^ These findings also support the importance of characteristic impedance (Zc) and aortic compliance, since both are highly dependent on vessel diameter (particularly Zc) and strongly influence PP for any given flow rate and/or stroke volume.^3,24,25^ However, our bidirectional MR findings showed that both increased genetically-predicted AscAoD was associated with decreased PP, and that an increased genetically-predicted PP was associated with decreased AscAoD, thus supporting a possible bidirectional causal association, rather than purely a hemodynamic effect of smaller ascending aortic diameter on aortic compliance and impedance as above. The primary mechanism by which an increased PP can lead to a smaller aortic diameter is unknown, but may involve extracellular matrix protein alterations in response to exaggerated cyclic variations in aortic wall stress. This mechanism may ultimately protect against aortic dilation, however it remains speculative and requires further study. Alternatively, our bidirectional findings may be explained by a shared genetic etiology due to traits influencing both aortic stiffness/diameter as well as BP.

The overall findings of our study may have implications for the prevention of thoracic aortic dilation. The current 2017 American Heart Association/American College of Cardiology and 2018 European Society of Cardiology/European Society of Hypertension guidelines on hypertension among adults at increased risk of cardiovascular disease suggest strong recommendations to prevent cardiovascular events, however there are limited data supporting the principle that BP control can prevent thoracic aortic dilation.^26^ Similarly, the 2010 American Heart Association/American College of Cardiology Foundation guidelines on diagnosis and management of thoracic aortic disease notes that tight BP control is the mainstay of medical treatment once a thoracic aortic aneurysm is diagnosed.^27^ Our MR study provides evidence of a causal effect of increased BP on ascending thoracic aortic dilation. In the absence of large, randomized trials of BP control focused on thoracic aorta-specific outcomes, our results are generally consistent with the notion that a lower blood pressure may limit aortic enlargement. Nevertheless, the quantitative impact of SBP, the usual BP target in current practice, was very small (point estimate = 0.0041 mm/mmHg). This was due to the discordant apparent causal effect of MAP and PP on AscAoD. In our multivariable MR analyses, both genetically-predicted reduced PP (β estimate = -0.2513 mm/mmHg increase) and genetically-predicted increased MAP (β estimate = 0.2624 mm/mmHg) were independently associated with AscAoD dimension. These discordant effects largely cancel out for SBP (which increases with increasing MAP and PP) but are additive for DBP (which increases with increasing MAP but decreases with increasing PP). As a consequence, genetically-predicted DBP exhibited the quantitatively largest causal association with AscAoD in our primary analyses. These results raise suggest that DBP may be a better target for BP control in this setting. However, this hypothesis should be studied further, ideally in randomized controlled trials. It should also be noted that further research is warranted to consider possible at-risk subpopulations where tighter BP control is indicated, or AscAoD thresholds above which empiric BP treatment would be indicated. Our MR study also raises new questions and avenues for future research regarding the interplay of PP, MAP and AscAoD, and the mechanism that drives the inverse relationship between the two.

This study has several limitations. First, whereas this secondary analysis using summary statistics from MVP was highly consistent with the primary analysis for SBP and PP, the results for DBP were not significant and demonstrated a high level of variance. This particular GWAS of BP identified 76 known and four novel genome wide significant SNPs associated with DBP compared to 216 known and 124 novel for SBP, and 208 known and 123 novel for PP, respectively, limiting the associated variants for DBP.^14^ Specifically, the identified novel SNPs had smaller effects on DBP (0.14 mmHg per allele) compared to SBP and PP (0.24 and 0.18 mmHg per allele, respectively). Additionally, sentinel SNPs at independent loci explained 1.06% of total variance in DBP compared to 3.56% and 3.72% of total variance for SBP and PP. These differences may reflect alterations in the phenotypic characteristics or controls utilized in the MVP GWAS, or how particular BP measurements were assessed among patient populations. Second, although we considered trans-ancestry studies of BP and AscAoD, the underlying populations were primarily composed of UKBB participants of European ancestry. Further study among populations of diverse ancestry will be critical to improve the generalizability of our results. Additional work should investigate the specific biologic pathways that govern the relationships between ascending thoracic aortic size and BP, including investigating the effect of comorbid conditions such as type II diabetes, which observationally has been shown to be protective against TAA,^28,29^ and hypercholesterolemia which has been shown to possibly contribute to TAA development.^30,31^ Finally, while our MR results were statistically robust when using MR methods that make different assumptions about the presence of pleiotropy (weighted median, inverse variance weighted, MR Egger), and when testing for the presence of reverse-causality via MR-Steiger, these methods are not perfect for attribution of causality.^13^

Overall, our findings are consistent with a causal effect of BP on AscAoD. We also found strong evidence of an inverse independent relationship between PP and AscAoD, which may be a priority for future investigation in susceptibility to thoracic aortic dilation. Finally, we found evidence consistent with a causal relationship between a higher AscAoD and lower SBP and PP, as well as a higher DBP, consistent with the role of aortic diameter as a determinant or aortic pulsatile hemodynamic properties.

## Supplementary Material

Dataset

Graphic Abstract

Supplemental Material

## Highlights

- Although observational studies have linked blood pressure (BP) to thoracic aortic aneurysm formation and growth, the relationship between BP and thoracic aortic diameter remains incompletely understood
- In this Mendelian randomization study, increases in BP as proxied by systolic BP, diastolic BP, and mean arterial pressure were robustly associated with increased ascending thoracic aortic diameter
- Genetic proxies for pulse pressure were inversely related to ascending thoracic aortic diameter
- Meanwhile, genetic proxies for ascending thoracic aortic diameter were inversely associated with systolic BP and pulse pressure, but positively associated with diastolic BP
- Overall, this study provides evidence consistent with a causal association between blood pressure and ascending thoracic aortic diameter
