# Supplementary figures and images for: Relationship between ascending thoracic aortic diameter and blood pressure, a Mendelian randomization study
Source: Arterioscler Thromb Vasc Biol. Author manuscript; Available in PMC 2023 Feb 1. (PMC7614108; doi:10.1161/ATVBAHA.122.318149)

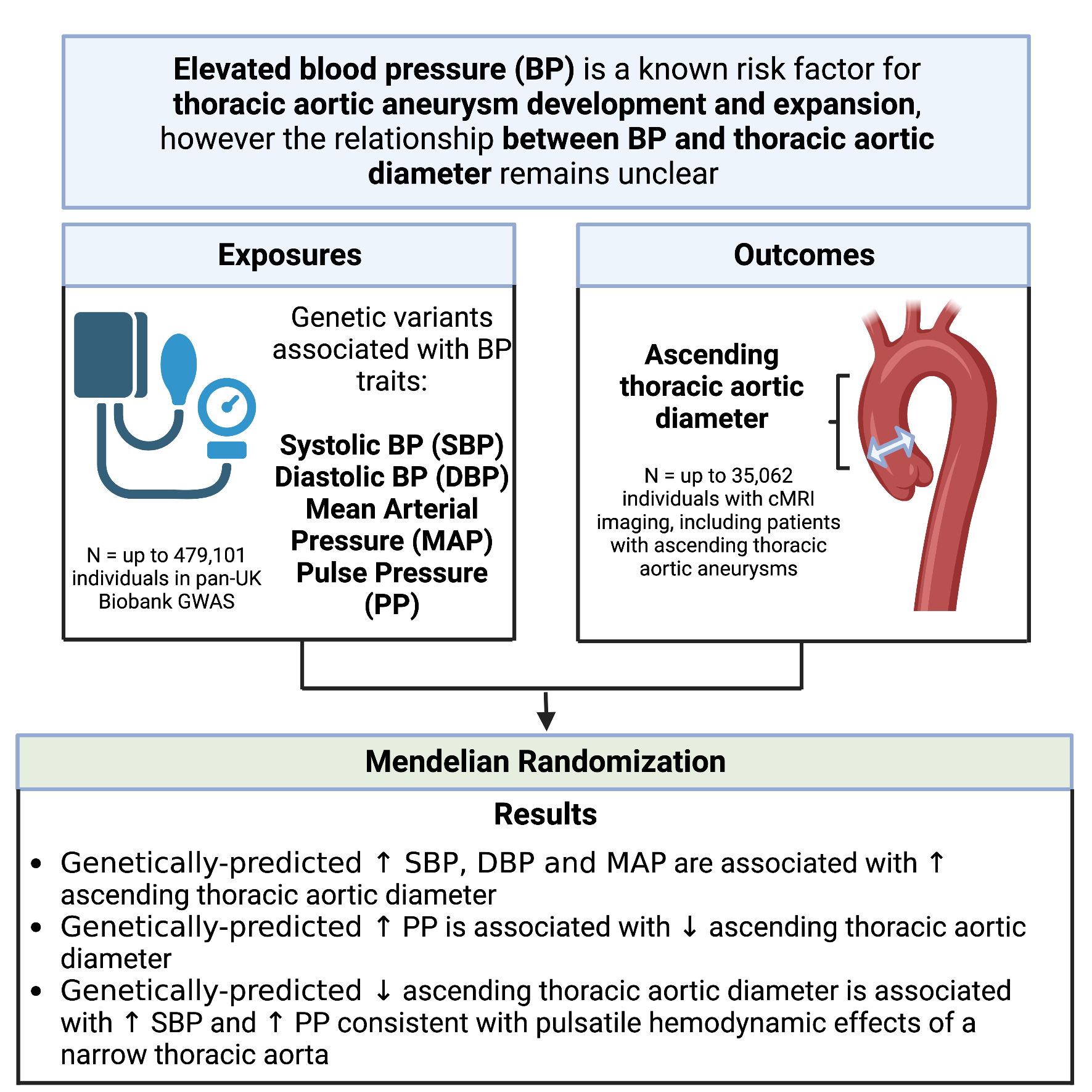

Supplement: Graphic Abstract [file EMS158829-supplement-Graphic_Abstract.jpg]
