# Supplementary material for: Relationship between ascending thoracic aortic diameter and blood pressure, a Mendelian randomization study
Source: Arterioscler Thromb Vasc Biol. Author manuscript; Available in PMC 2023 Feb 1. (PMC7614108; doi:10.1161/ATVBAHA.122.318149)
Supplement: Supplemental Material [file EMS158829-supplement-Supplemental_Material.pdf]

SUPPLEMENTAL MATERIALS: Relationship between ascending thoracic aortic diameter and blood pressure, a Mendelian randomization study

Authors: John DePaolo, MD, PhD,[1] Michael G. Levin,[2] MD, Catherine Tcheandjieu, PhD,[3,4] James Priest, MD,[5] Dipender Gill, MD, PhD,[6-7] Stephen Burgess, PhD,[8,9] Scott M. Damrauer, MD,[1,10,11] Julio A. Chirinos, MD, PhD[2]

Affiliations:
[1] Department of Surgery, Perelman School of Medicine, University of Pennsylvania, Philadelphia, PA, USA.
[2] Division of Cardiovascular Medicine, Department of Medicine, Perelman School of Medicine, University of Pennsylvania, Philadelphia, PA, USA.
[3] Gladstone Institute of Data Science and Biotechnology, Gladstone Institutes, San Francisco, CA, USA.
[4] Department of Epidemiology and Biostatistics, University of California San Francisco, San Francisco, CA, USA
[5] Department of Pediatrics – Division of Pediatric Cardiology, Stanford University School of Medicine, Stanford, CA, USA.
[6] Chief Scientific Advisor Office, Research and Early Development, Novo Nordisk, Copenhagen, Denmark.
[7] Department of Epidemiology and Biostatistics, School of Public Health, Imperial College London, UK.
[8] MRC Integrative Epidemiology Unit, University of Bristol, Bristol, UK.
[9] Department of Public Health and Primary Care, University of Cambridge, Cambridge, UK.
[10] Corporal Michael Crescenz VA Medical Center, Philadelphia, PA, USA.
[11] Department of Genetics, Perelman School of Medicine, University of Pennsylvania, Philadelphia, PA, USA.

Figure S1:

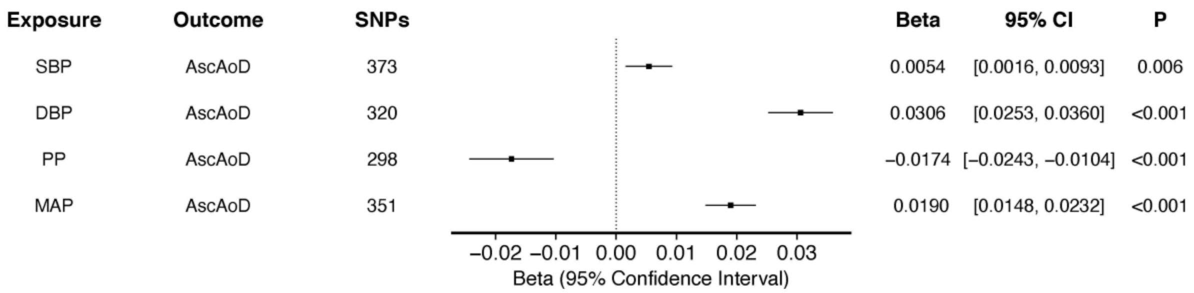

Figure S1. Association between genetically-predicted blood pressure traits and ascending thoracic aortic diameter by univariate two-sample MR using IVW analysis among up to 479,101 participants in the UK Biobank with BP traits and a cohort of up to 42,518 participants who underwent MRI for thoracic diameter evaluation, excluding aortas > 5 cm in diameter. Genetically-predicted blood pressure as proxied by SBP (β estimate = 0.0054 mm/mmHg, 95% CI 0.0016 to 0.0093, p = 0.006), DBP (β estimate = 0.0306 mm/mmHg, 95% CI 0.0253 to 0.0360, p < 0.001), and MAP (β estimate = 0.0190 mm/mmHg, 95% CI 0.0148 to 0.0232, p < 0.001) have a positive association with increased AscAoD by univariate two-sample MR using IVW analysis when excluding all aortas > 5 cm in diameter. PP (β estimate = -0.0174, 95% CI -0.0243 to -0.0104, p < 0.001) has a negative association with AscAoD.

Figure S2:

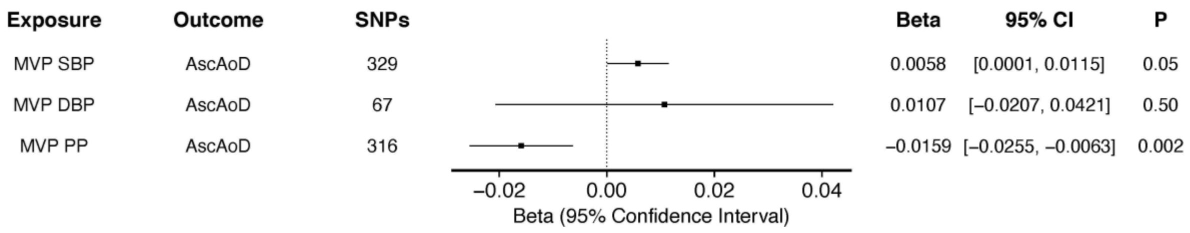

Figure S2. Association between genetically-predicted blood pressure traits and ascending thoracic aortic diameter by univariate two-sample MR using IVW analysis among up to 318,891 participants in the MVP with BP traits and a cohort of up to 35,062 who underwent MRI for thoracic diameter evaluation. Genetically-predicted blood pressure as proxied by SBP (β estimate = 0.0058 mm/mmHg, 95% CI 0.0001 to 0.0115, p = 0.05) among participants in MVP has a positive association with increased ascending thoracic aortic diameter by univariate two-sample MR using IVW analysis. PP (β estimate = -0.0159 mm/mmHg, 95% CI -0.0255 to -0.0063, p = 0.002) has an inverse causal relationship with AscAoD. DBP (β estimate = 0.0107 mm/mmHg, 95% CI -0.0207 to 0.0421, p = 0.5) demonstrated statistically insignificant results.

Major Resources Table

In order to allow validation and replication of experiments, all essential research materials listed in the Methods should be included in the Major Resources Table below. Authors are encouraged to use public repositories for protocols, data, code, and other materials and provide persistent identifiers and/or links to repositories when available. Authors may add or delete rows as needed.

Animals (in vivo studies)

Species	Vendor or Source	Background Strain	Sex	Persistent ID / URL
N/A	N/A	N/A	N/A	N/A

Genetically Modified Animals

	Species	Vendor or Source	Background Strain	Other Information	Persistent ID / URL
**Parent - Male**	N/A	N/A	N/A	N/A	N/A
**Parent - Female**					

Antibodies

Target antigen	Vendor or Source	Catalog #	Working concentration	Lot # (preferred but not required)	Persistent ID / URL
N/A	N/A	N/A	N/A	N/A	N/A

DNA/cDNA Clones

Clone Name	Sequence	Source / Repository	Persistent ID / URL
N/A	N/A	N/A	N/A

Cultured Cells

Name	Vendor or Source	Sex (F, M, or unknown)	Persistent ID / URL
N/A	N/A	N/A	N/A

Data & Code Availability

Description	Source / Repository	Persistent ID / URL
Dataset – Complete Supplementary Data Tables	Excel spreadsheet included within manuscript submission	N/A

Other

Description	Source / Repository	Persistent ID / URL
N/A	N/A	N/A

ARRIVE GUIDELINES

The ARRIVE guidelines (https://arriveguidelines.org/) are a checklist of recommendations to improve the reporting of research involving animals. Key elements of the study design should be included below to better enable readers to scrutinize the research adequately, evaluate its methodological rigor, and reproduce the methods or findings.

Study Design

**Groups**	**Sex**	**Age**	**Number (prior to experiment)**	**Number (after termination)**	**Littermates (Yes/No)**	**Other description**
Group 1 (Control)	N/A	N/A	N/A	N/A	N/A	N/A
Group 2	N/A	N/A	N/A	N/A	N/A	N/A
Add more if needed	N/A	N/A	N/A	N/A	N/A	N/A

Sample Size: Please explain how the sample size was decided Please provide details of any a *prior* sample size calculation, if done.

N/A

Inclusion Criteria

N/A

Exclusion Criteria

N/A

Randomization

N/A

Blinding

N/A
